# The Tryptophan Paradox: From Microbiome-Mediated Homeostasis to Tumor-Driven Immune Escape

**DOI:** 10.3390/ijms27104296

**Published:** 2026-05-12

**Authors:** Alexis Cho, Chunjing Wu, George Theodoropoulos, Manojavan Nagarajan, Adeline M. Murphy, Karli F. Heller, Niramol Savaraj, Theodore J. Lampidis, Medhi Wangpaichitr

**Affiliations:** 1 Department of Veterans Affairs, Miami VA Healthcare System, Miami, FL 33125, USA; alexischo33@gmail.com (A.C.); chunjing.wu@va.gov (C.W.); gxt25@miami.edu (G.T.); mxn816@miami.edu (M.N.); amm7470@miami.edu (A.M.M.); kfh34@miami.edu (K.F.H.); niramol.savaraj@va.gov (N.S.); 2 Sylvester Comprehensive Cancer Center, University of Miami, Miami, FL 33136, USA; tlampidi@med.miami.edu; 3 South Florida VA Foundation for Research and Education, Miami, FL 33125, USA; 4 Department of Cell and Systems Biology, University of Miami, Miami, FL 33136, USA; 5 Department of Surgery, Division of Thoracic Surgery, University of Miami, Miami, FL 33136, USA

**Keywords:** tryptophan, kynurenine, aryl hydrocarbon receptor, microbiome, immunometabolism

## Abstract

Tryptophan (Trp) metabolism sits at the intersection of nutrition, the microbiome, mucosal immunity, and tumor adaptation. The broad observation that microbial indoles can support barrier function, whereas tumors exploit kynurenine-pathway metabolism to suppress immunity, is already established in publications. The specific contribution of this review is to organize that literature into a context- and network-based translational framework. Rather than treating indoleamine 2,3-dioxygenase 1 (IDO1) as a single bottleneck, we frame tumor Trp metabolism as a compensatory system linking IDO1, tryptophan 2,3-dioxygenase (TDO2), interleukin-4-induced gene 1 (IL4I1), amino-acid transport, amino-acid stress sensing, and downstream aryl hydrocarbon receptor (AHR) signaling. In healthy tissue, especially the gut, dietary Trp and microbiota-derived indoles can promote epithelial integrity, interleukin-22 (IL-22)-associated programs, and mucosal restraint. In tumors, the same substrate pool is redirected toward Kynurenine, kynurenic acid, indole-3-pyruvate, and related catabolites that impair cytotoxic lymphocytes, expand regulatory T-cell (Treg) and suppressive myeloid compartments, and reinforce invasion and treatment resistance. We also argue that the potential metabolite biomarker interpretation should be context-dependent. Finally, we propose a clinical-context–specific framework for intervention. Dietary and microbiome-based strategies may be most effective in prevention, premalignant states, or supportive care, whereas established cancers are more likely to require biomarker-guided targeting of tumor-associated catabolic pathways and convergent signaling mechanisms. The “paradox” is therefore not that Trp changes chemistry across settings, but that the same nutrient is routed through different cellular contexts, enzymes, ligands, and cell states.

## 1. Introduction

Tryptophan metabolism lies at a crossroads connecting nutrient supply, microbial ecology, immune programming, and nicotinamide adenine dinucleotide (NAD) biosynthesis. In cancer biology, interest in this axis first centered on indoleamine 2,3-dioxygenase 1 (IDO1), an interferon-gamma (IFNg)-inducible extrahepatic heme enzyme that catalyzes the initial oxidative step of Trp catabolism and can suppress T-cell and natural killer (NK)-cell function through a combination of local Trp restriction and immunoregulatory catabolite production [1,2,3]. That view has since broadened. Tryptophan 2,3-dioxygenase (TDO2), the physiologic hepatic dioxygenase, can be constitutively active in tumors or stromal cells, and interleukin 4-induced-1 (IL4I1), a secreted L-amino-acid oxidase enriched in antigen-presenting and myeloid compartments, provides a parallel route to aryl hydrocarbon receptor (AHR)activation via Trp metabolites [4,5].

Several recent reviews have already summarized the general biology of Trp catabolism, AHR signaling, and kynurenine-pathway therapeutics [6,7,8,9,10]. The value of the present review is therefore not to relabel the field, but to synthesize it in a way that is more directly useful for translational decision-making. We use the phrase “tryptophan paradox” as shorthand for a compartment-dependent reversal in biologic meaning: in nonmalignant tissue, especially along the gut barrier, Trp-derived indoles can stabilize mucosal homeostasis, whereas in tumors the same substrate is rerouted into suppressive and tumor-promoting programs.

Our emphasis is on two interconnected concepts that are often considered separately. First, tumor Trp catabolism functions as a compensatory network rather than a pathway driven solely by IDO1. Second, biomarkers and therapeutic strategies should be aligned with disease and tissue context, rather than applied uniformly across prevention, early disease, and advanced cancer.

The review is organized around that framework. Section 2 revisits the underlying biology, including IL4I1, general control nonderepressible 2 kinase (GCN2)-dependent stress sensing, L-type amino acid transporter 1 (LAT1)-mediated transport, and metabolite-specific receptor pharmacology beyond kynurenine (Kyn) alone. Section 3 expands the microbiome discussion to include both barrier biology and the immunotherapy literature. Section 4 then interprets biomarker data across selected tumor settings with explicit attention to study design, specimen type, and model system. Section 5 translates these observations to therapy, including a more cautious interpretation of the previous clinical trial and the implications of pathway compensation.

## 2. Mechanistic Basis of the Paradox

### 2.1. Physiologic Trp Fates

Mammalian cells cannot synthesize the indole ring de novo, so Trp availability depends on diet, intestinal absorption, systemic distribution, and competition among host tissues and the microbiota (Xue et al., 2023 [11]). Under physiologic conditions, Trp is partitioned among several major fates: protein synthesis through tryptophanyl-tRNA synthetase, serotonin and melatonin synthesis through tryptophan hydroxylases, Kyn-pathway metabolism with downstream contribution to de novo NAD biosynthesis, and microbial conversion in the intestinal lumen to indoles such as indole-3-acetic acid (IAA), indole-3-aldehyde (IAld), indole-3-propionate (IPA), and indole-3-lactate (ILA) [11,12,13].

**Figure 1:**
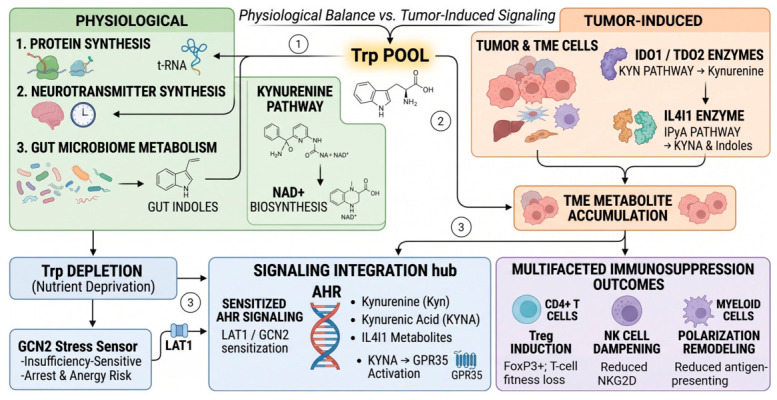
Trp metabolism paradox. Under physiological conditions (1), Trp supports protein synthesis, neurotransmitter production, gut microbiome-derived indole generation, and Kyn-pathway contribution to NAD biosynthesis. In tumors (2), Trp is redirected by tumor and TME cells through IDO1/TDO2-mediated Kyn production and IL4I1-mediated generation of indole-3-pyruvate, kynurenic acid, and related indoles. Accumulation of these metabolites (3), together with Trp depletion (3), GCN2 stress sensing, LAT1-mediated uptake, and AHR/GPR35 signaling, promotes regulatory T-cell (Treg) induction, NK-cell dampening, myeloid remodeling, and broader immunosuppressive outcomes. Created in BioRender. Wangpaichitr, M. (2026) https://BioRender.com/h6qu5op (accessed on 7 May 2026).

Tumor metabolic reprogramming shifts the balance of these outcomes, but not uniformly across cancers. IDO1 is often induced in tumor or myeloid cells by inflammatory cytokines such as IFNg, whereas TDO2 may be constitutively expressed in tumor cells or stromal populations and is particularly relevant in tissues where the enzyme is already present at physiologic levels, including the liver [14]. In contrast, IL4I1 is secreted rather than intracellular, is associated with antigen-presenting and suppressive myeloid states and produces a broader spectrum of metabolites than the canonical Kyn-pathway [4,5]. The functional consequence is not simply Trp consumption. Rather, the tumor microenvironment (TME) accumulates ligands and co-signals that bias CD4+ T-cell differentiation toward FoxP3+ regulatory T-cell (Treg) states, dampen natural killer group 2 member D receptor (NKG2D) expression, and remodel myeloid polarization [1,2,3] (Figure 1).

It is important, however, to avoid presenting every compensatory observation as universal. Selective IDO1 blockade has been shown most clearly in cisplatin-resistant non-small cell lung cancer models from our group to trigger TDO2 upregulation and preserve Kyn production [15]. That is a useful model for network compensation, but it should be interpreted as the best-documented example in one tumor context rather than a proven pan-cancer mechanism.

### 2.2. IL4I1 as a Parallel Escape Route for Trp

IL4I1 encodes a secreted L-amino-acid oxidase originally characterized in immune cells, especially antigen-presenting and myeloid compartments [16]. Within TME, IL4I1 can be produced by dendritic cells, macrophages, and immunosuppressive stromal populations, and tumor-cell expression has also been reported in specific contexts [4,5]. In human head and neck cancer-derived mesenchymal stromal cells, IL4I1 expression contributed directly to suppression of T-cell proliferation, supporting the idea that the pathway is not restricted to canonical professional immune cells [17].

Its metabolic output differs from that of IDO1 or TDO2. Whereas IDO1 and TDO2 directly initiate Kyn production from Trp, IL4I1 catalyzes the oxidative deamination of aromatic amino acids and can convert Trp to indole-3-pyruvate (IPyA), which in turn gives rise to indole derivatives and kynurenic acid (KYNA), thereby expanding the repertoire of potential AHR ligands [4]. This difference matters because “Kyn pathway activation” is often discussed as if a single catabolite were responsible for all downstream effects (Figure 1). Preclinical studies support an IL4I1-mediated immune-escape pathway in which IL4I1-derived metabolites activate AHR, promote tumor progression, including in breast, lung, and liver cancers, and remain active even when upstream IDO1 activity is constrained [4]. The field is still earlier than the IDO1 literature, and direct IL4I1-selective inhibition is not yet clinically mature. Accordingly, the review advocating network-level rather than single-node intervention should explicitly recognize that IL4I1 is not merely ancillary to IDO1 and TDO2 but represents a mechanistically distinct branch of immunosuppressive tryptophan metabolism.

### 2.3. Beyond AHR Alone: GCN2, Transport, and Metabolite-Specific Signaling

AHR is a major signaling hub for the Trp metabolites, but it is not the only relevant sensor. Trp depletion can be detected through the integrated stress response, particularly by the kinase GCN2, which is activated by amino-acid insufficiency and uncharged tRNA. In an early mechanistic study using murine plasmacytoid dendritic cells and Gcn2-deficient T cells, Munn and colleagues showed that GCN2 was required for IDO-mediated proliferative arrest and anergy induction [18]. In experimental B16 melanoma, Sonner et al. argued that GCN2 did not mediate suppression of antitumor T-cell responses by Trp catabolism in the way originally proposed [19]. In a separate TCR-transgenic mouse system, Van de Velde et al. showed that GCN2 was required for normal CD8+ T-cell proliferative fitness and trafficking, independent of simple environmental amino-acid sensing [20]. Together, these studies argue against a one-faceted explanation in which Trp depletion uniformly shuts down immunity through GCN2 alone.

Recent work helps reconcile these observations. In IDO/TDO overexpressed cells, multiple human tumor cell lines, and murine naive CD4+ T-cell differentiation assays, Solvay et al. showed that Trp depletion sensitizes the AHR pathway by increasing AHR expression and GCN2/LAT1-dependent Kyn uptake, thereby potentiating Treg induction even when Trp catabolites are relatively weak agonists under baseline conditions [21]. The emerging view is that depletion and catabolite production act cooperatively: nutrient deprivation reshapes receptor abundance, transport capacity, and transcriptional state, thereby heightening cellular sensitivity to ligands that would otherwise be functionally insufficient.

Transport is another underappreciated layer that exerts an impact on this Trp metabolism path. LAT1/SLC7A5 imports Trp but also Kyn and other large neutral amino acids, linking extracellular nutrient composition to intracellular signaling [22,23]. That makes LAT1 attractive as a metabolic target in cancers with high transport dependence, but it also creates a risk: the same transporter supports effector lymphocytes, so indiscriminate blockade could impair antitumor immunity as well as tumor growth [24]. For the same reason, transporter molecular expression should be thought of as a context-setting variable rather than a purely tumor-intrinsic marker.

Downstream metabolites do not engage identical receptors. While Kyn is the best-characterized ligand for AHR, KYNA displays distinct receptor pharmacology. Wang et al. identified KYNA as a ligand for G protein-coupled receptor 35 (GPR35), and DiNatale et al. further demonstrated that KYNA can also activate the human AHR [25,26]. Xanthurenic acid and other downstream metabolites likely add additional receptor diversity that is still incompletely mapped in cancer. Lumping all of these molecules into “Kyn signaling” obscures a key translational issue: the output of the pathway depends not only on flux, but also on which metabolite accumulates in which cell type.

### 2.4. AHR Output Is Context-Determined, Not Intrinsically Homeostatic or Malignant

NAD biology represents another layer. Because the Kyn pathway contributes to de novo NAD synthesis, it is tempting to describe tumors as broadly dependent on Trp catabolism for NAD supply. Some tumors exploit Kyn-pathway metabolites for bioenergetic or redox support, but NAD metabolic dependency is strongly shaped by tissue context and by the relative availability of salvage pathways such as nicotinamide phosphoribosyltransferase (NAMPT) and nicotinate phosphoribosyltransferase NAPRT [27,28]. Accordingly, not all tumors are expected to depend strongly on de novo NAD synthesis from Trp; rather, NAD-related vulnerabilities are likely to emerge in specific contexts where enhanced Trp catabolism coincides with limited NAD salvage capacity, creating a restrictive metabolic state that constrains redox balance and nucleotide homeostasis.

**Figure 2:**
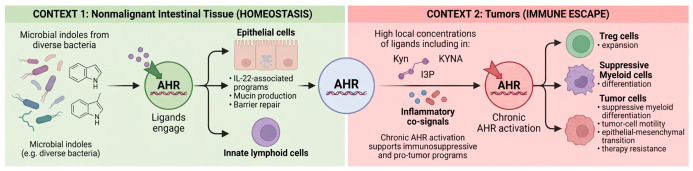
Context-dependent AHR signaling. In healthy intestine (green box), microbiota-derived indoles activate AHR in epithelial and innate lymphoid cells to support IL-22–dependent barrier homeostasis. In tumors (red box), inflammatory signals and accumulation of Kyn-pathway and related metabolites drive chronic AHR activation, promoting immunosuppression, tumor plasticity, and treatment resistance. Created in BioRender. Wangpaichitr, M. (2026) https://BioRender.com/reooh8i (accessed on 7 May 2026).

AHR thus functions as a context-determining transcription factor that integrates metabolic and environmental signals, rather than acting as a fixed downstream effector. In nonmalignant intestinal tissue, microbial indoles engage AHR in epithelial cells and innate lymphoid populations to support interleukin-22 (IL-22)-associated programs, mucin production, and barrier repair [12,13]. In tumors, high local concentrations of Kyn, KYNA, I3P, and inflammatory co-signals promote chronic AHR activation that supports Treg expansion, suppressive myeloid differentiation, tumor-cell motility, epithelial–mesenchymal transition, and therapy resistance [4,9]. The same receptor can therefore maintain tissue homeostasis in one context while promoting immune escape in another. This is not because AHR itself changes function, but because ligand identity, concentration, temporal exposure, cell state, and tissue context vary together (Figure 2).

## 3. The Microbiome Axis: From Barrier Homeostasis to Immunotherapy Modulation

The microbiome warrants more thorough consideration than it typically receives in pathway-centered reviews, as it represents a prominent context in which Trp-derived signals are not inherently immunosuppressive. Commensal bacteria across genera, including *Lactobacillus*, *Clostridium*, *Bacteroides*, *Ruminococcus*, and *Peptostreptococcus*, participate in Trp conversion to indoles and related molecules that engage epithelial and innate immune pathways [12,13,29]. In Cell–Host and Microbe, Wlodarska et al. showed that indole-3-acrylic acid (IAcrA) produced by Peptostreptococcus species improved barrier function and dampened inflammatory signaling, providing a concrete example of metabolite-level rather than taxon-level benefit [30]. Scott et al. further demonstrated that microbial Trp metabolites regulate gut barrier function through AHR, strengthening the mechanistic link between commensal chemistry and epithelial defense [13].

In a 2024 study, Sinha et al. showed that dietary fiber redirected microbial Trp metabolism through metabolic interactions within the gut microbiota, highlighting that the relevant exposure is not simply more Trp, but the ecological conditions under which Trp is processed [29]. That point matters clinically because nutritional interventions are often described too broadly. A high-fiber or Mediterranean-style pattern may favor beneficial indole output in some individuals, but the downstream effect depends on microbiome composition, host inflammation, and the disease compartment being targeted (Diet prevention is further discussed in Section 5.3).

The cancer immunotherapy literature makes the microbiome story even more relevant. In two landmark Science studies, Gopalakrishnan et al. in melanoma and Routy et al. in epithelial tumors reported associations between gut microbiome composition and response to programmed cell death protein 1 (PD-1)-based therapy [31,32]. Those studies were correlative but mechanistically important because they connected microbial ecology with checkpoint efficacy in humans. Davar et al. later moved beyond association by showing in a phase 1 trial that responder-derived fecal microbiota transplantation (FMT) plus anti-PD-1 therapy induced clinical benefit in a subset of PD-1-refractory melanoma patients and was accompanied by immunologic and metabolomic reprogramming of the TME [33]. More recently, Bender et al. reported in Cell that an intratumoral *Lactobacillus reuteri*-derived dietary Trp metabolite facilitated immune checkpoint inhibitor activity, suggesting that microbe-dependent Trp metabolism can shape not only the gut compartment but also the tumor itself [34].

**Figure 3:**
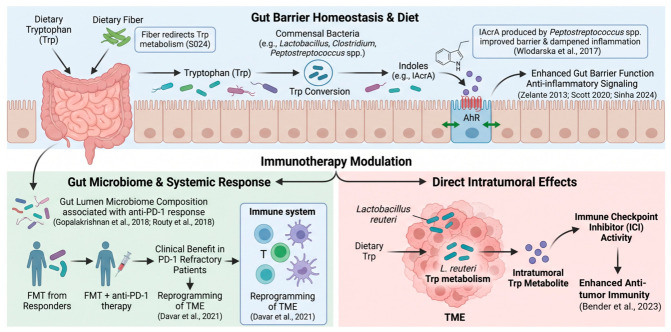
Microbiome-mediated Trp metabolism in barrier function and immunotherapy. Dietary Trp and fiber shape commensal production of indole metabolites that activate epithelial AHR, strengthen gut barrier function, and dampen inflammation (blue box). Microbiome composition and intratumoral *Lactobacillus reuteri*-derived Trp metabolites can further modulate anti-PD-1 response, TME reprogramming, and immune checkpoint inhibitor activity [12,13,29,30,31,32,33,34] (green and red boxes). Created in BioRender. Wangpaichitr, M. (2026) https://BioRender.com/muukhmj (accessed on 7 May 2026).

At the same time, not every Trp-metabolizing microbe is beneficial in every disease. Montgomery et al. showed that *Lactobacillus. reuteri* Trp metabolism promoted susceptibility to central nervous system autoimmunity in a murine experimental autoimmune encephalomyelitis model [35]. That study is a useful corrective to simplistic language such as “indoles are protective” or “probiotics are beneficial.” Microbial Trp metabolism is biologically powerful precisely because it is context-dependent. In cancer, the most balanced interpretation is that microbiome-directed strategies may modulate mucosal immune tone and, in selected contexts, influence responsiveness to immunotherapy; however, they should be framed as hypothesis-driven, disease-context-specific interventions rather than nonspecific adjuncts. Figure 3 summarizes the microbiome-Trp axis from gut barrier homeostasis to immunotherapy modulation.

## 4. Biomarkers and Tumor Contexts

The circulating Kyn/Trp ratio is the most commonly used readout of tumor Trp catabolic activity, yet it is intrinsically blind to biological context. This metric integrates hepatic metabolism, systemic inflammation, renal clearance, corticosteroid exposure, dietary intake, and whole-body enzyme activity, while most published studies do not pair blood measurements with tumor-level metabolomics, spatial transcriptomics, or immune-cell phenotyping [36,37]. Accordingly, plasma Kyn/Trp should be described as an associative systemic biomarker. It can correlate with risk or outcome in specific cohorts, but it should not be elevated to the status of a validated tumor-specific surrogate without paired tissue data. The spatially resolved interpretation of the Trp axis is summarized in Table 1.

### 4.1. Non-Small Cell Lung Cancer (NSCLC)

NSCLC remains one of the more informative translational examples, but the limitations of the literature need to be kept in view. Serum Kyn/Trp has been reported as higher in lung cancer patients than in controls and has been associated with advanced stage and disease progression [37,38]. In prospective epidemiology, Chuang et al. analyzed pre-diagnostic blood specimens from the European Prospective Investigation into Cancer and Nutrition (EPIC) and found that higher Kyn/Trp was associated with increased lung cancer risk, especially for squamous histology [39]. These are meaningful observations, but they do not establish tumor-local pathway dominance because the measurements are systemic and pre-diagnostic. The same caution applies to treatment response. In a retrospective 104-patient NSCLC radiotherapy cohort, Zhu et al. reported that higher pretreatment Kyn/Trp was associated with worse progression-free survival and that post-treatment dynamics also tracked outcome [40]. This supports the ratio as a pharmacodynamic or prognostic correlate in that specific cohort, not as a universally validated biomarker.

### 4.2. Glioblastoma

Glioblastoma adds a distinct set of interpretive constraints because the central nervous system has unique immune, metabolic, and neuroactive ligand biology. Kynurenine-pathway metabolites in the brain can affect not only immune regulation but also neuromodulatory and neuroinflammatory processes. In a 108-patient newly diagnosed GBM cohort, Jacquerie et al. found that high tumor expression of IDO1, IDO2, TDO2, and AHR was associated with poorer survival, supporting tumor-compartment profiling rather than sole reliance on plasma measurements [41]. A later study from the same group suggested that an IDO2-AHR axis may be especially relevant in GBM [41]. That finding is important and hypothesis-generating, but the field should resist turning it into a definitive explanation for why first-generation IDO1 inhibitors underperformed clinically. At present, the safest conclusion is that GBM likely requires intratumoral or tumor-adjacent readouts and that pathway dominance may differ from peripheral tumor sites.

### 4.3. Hepatocellular Carcinoma

Hepatocellular carcinoma illustrates why tissue physiology matters. Because TDO2 is a normal hepatic enzyme, its presence in liver tissue is not intrinsically pathological. What matters is dysregulated expression within malignant or stromal compartments, the accompanying metabolite context, and the immune consequences. Hoffmann et al. identified TDO2 expression in human HCC cells and in intratumoral pericytes across cancers, while Li et al. and Liu et al. linked high TDO2 activity to epithelial–mesenchymal transition, Wnt5a signaling, migration, invasion, and poor prognosis in HCC models and human cohorts [14,42,43]. Some experimental reports suggest context-dependent growth-restraining effects of TDO2, but the dominant clinical signal remains that high tumoral TDO2 marks a more aggressive, immunosuppressive disease state. HCC therefore cautions against a common analytic mistake: assuming that a physiologic baseline enzyme cannot be oncogenic simply because it is native to the tissue.

### 4.4. Colorectal Cancer

Colorectal cancer brings both sides of the paradox into the same organ system. On the one hand, microbiota-derived indoles contribute to barrier integrity and restraint of mucosal inflammation, processes that are plausibly protective in early colorectal carcinogenesis. On the other hand, tumor-associated IDO1 expression, particularly at the invasion front, has long been associated with reduced T-cell effectiveness, metastasis, and poorer survival [44,45]. Zhang et al. further identified an IDO1/CD8-high colon cancer subtype in which immune infiltration coexisted with functional suppression and poor outcome, emphasizing that cell abundance alone does not equal effective antitumor immunity [46]. In a large FOCUS Consortium analysis of 2102 stage I-III CRC patients, circulating kynurenine-pathway metabolites, including Kyn and 3-hydroxykynurenine, were associated with all-cause mortality [47]. That is valuable prognostic evidence, but like the NSCLC data, it should still be interpreted as systemic and research-grade rather than standard of care. Preclinical evidence that IDO1 inhibition can enhance anti-PD-1 efficacy in microsatellite-stable CRC further supports the pathway as actionable, but not yet clinically resolved [48].

## 5. Therapeutic Translation

### 5.1. Limitations of IDO1-Only Therapeutic Strategies

The negative result of ECHO-301/KEYNOTE-252 should not be simplified to “the Trp pathway failed.” Epacadostat is an oral selective IDO1 inhibitor, whereas pembrolizumab is an antibody against PD-1. The combination was rational because early phase 1/2 ECHO-202/KEYNOTE-037 data in advanced melanoma appeared promising: among 54 response-evaluable patients, the objective response rate was 56%, and among treatment-naive patients treated at the recommended phase 2 dose of epacadostat 100 mg twice daily plus pembrolizumab 200 mg every 3 weeks, the objective response rate was 60% [49]. This phase 1/2 likely contributed to the enthusiasm for the phase 3 trial in unresectable or metastatic melanoma [50].

Why, then, did the phase 3 strategy fail? Several explanations remain plausible and are not mutually exclusive. First, patients were not selected based on intratumoral IDO1 activity, which is Kyn metabolite accumulation, or AHR target-gene activation, thus highlighting the absence of validated biomarkers to identify pathway-dependent tumors. Second, the pharmacodynamic adequacy of intratumoral pathway suppression was uncertain. Third, melanoma in many patients may not have been dominantly driven by IDO1 even when immune evasion was clearly present. Fourth, network redundancy may have allowed continued suppressive signaling through TDO2, IL4I1, transport-dependent ligand uptake, or downstream AHR activation.

The failure of IDO1-only strategies argues not for abandoning the pathway, but for refining its therapeutic exploitation. Matching clinical interventions to pathway architecture, such as high intratumoral kynurenine signaling, strong AHR transcriptional output, or compensatory enzyme induction, may favor downstream or combination approaches over IDO1 monotherapy in unselected settings.

### 5.2. Toward a Network-Based Therapeutic Strategy

A network-based strategy can take several forms. One approach is to move downstream and target convergent signaling rather than a single upstream enzyme. Early-phase clinical development of AHR antagonists such as BAY 2416964 and IK-175 demonstrated that the molecule is druggable in humans, even though the most informative biomarker sets are still evolving. Another approach is to combine upstream blockade with better pharmacodynamic readouts, including paired tumor biopsies, tumor-metabolite profiling, and target-gene signatures rather than plasma Kyn/Trp alone. A third approach is to exploit context-dependent vulnerabilities in transport or NAD metabolism, but only with explicit consideration of immune fitness, as the same transporters and salvage pathways are required to sustain antitumor lymphocyte function.

Although IDO/TDO/IL4I1-directed strategies remain at an earlier stage of translational development, the key implication is clear: preservation of AHR-active signaling through alternative catabolites implies that approaches ignoring noncanonical metabolite measurements may miss critical network redundancy. Table 2 summarizes the major therapeutic concepts, representative examples, and practical caveats.

### 5.3. Diet and Microbiome Interventions: Prevention and Support Versus Established Disease

Diet and microbiome interventions warrant particularly rigorous evaluation. Nonetheless, in preventive, premalignant, or supportive-care settings, approaches that enhance diet quality, augment fermentable fiber availability, or promote indole-producing commensals remain biologically plausible, given their potential to reinforce epithelial barrier function and reduce inflammatory signaling.

Human evidence remains limited. However, in a case–control analysis of 3759 cancer cases and 2995 control subjects of whom 37 with pancreatic cancer, Luu et al. reported that higher dietary Trp intake was associated with lower pancreatic cancer risk, with an odds ratio of 0.51 (95% CI 0.29–0.92) for continuous intake and a significant trend across tertiles, but the sample size was small, and the confidence intervals were wide [51]. Although informative, these results remain hypothesis-generating and will require further clinical corroboration before informing practice.

In malignancy, augmenting substrate availability cannot be presumed beneficial. Nutritional or microbiome interventions during active treatment should be standardized, accompanied by metabolite-based monitoring, and interpreted in the tumor context rather than deployed empirically. Taken together, diet and microbiome modulation may be best suited to prevention or barrier-supportive settings, whereas advanced tumors likely require direct targeting of tumor-associated catabolism and downstream signaling.

## 6. Limitations and Future Directions

Several limitations still constrain near-term clinical application of these concepts. Much of the mechanistic literature remains preclinical, and the species- and model-dependence of key observations is not trivial. GCN2 biology, for example, differs across murine T-cell systems and cannot be summarized by a single depletion-centric narrative. Human nutrition and microbiome studies are often small, heterogeneous, and rarely paired with tumor measurements. Even in the biomarker literature, the widespread use of plasma Kyn/Trp as a tumor-specific assay continues to outpace the evidence supporting such a use.

Future study designs should integrate plasma, tumor, and stool or mucosal measurements; incorporate spatially resolved or single-cell readouts of enzyme expression and immune state spatially; and evaluate interventions matched to the dominant pathway signaling in each specific context. Prevention-oriented studies should prioritize barrier function, dietary modulation, and microbial metabolite output. In contrast, trials in established disease should interrogate whether the primary actionable driver is upstream amino-acid catabolism, transport dependence, alternative catabolite chemistry, or downstream AHR signaling. Engineered microbial strategies and pathway-informed combination therapies may ultimately render this axis more tractable, but only if benchmarked against biomarkers appropriate to the relevant biological compartment.

## 7. Conclusions

The tryptophan paradox in cancer reflects biological context rather than contradiction. In barrier tissues, particularly the gut, dietary tryptophan and microbiota-derived indoles support epithelial integrity and immune balance, whereas in established tumors the same substrate is frequently rerouted through IDO1, TDO2, IL4I1, transport pathways, and AHR signaling to drive immune suppression and therapeutic resistance. This context-dependent routing explains both the limitations of systemic biomarkers such as the Kyn/Trp ratio and the failure of IDO1-only inhibition in unselected populations. Progress will require context-specific, network strategies that integrate compartment-appropriate biomarkers with targeted intervention. Thus, the translational challenge is not simply to block Trp metabolism, but to define when, where, and through which cellular networks it shifts from homeostatic support to malignant advantage.

## Figures and Tables

**Table 1:** Compartment-specific interpretation of the tryptophan axis.

Compartment	Common Readouts	Interpretation of Readout	Major Confounders	Best Companion Measurement
Plasma or serum	Kyn, Trp, Kyn/Trp, broader kynurenine-pathway panel	Accessible systemic correlate of inflammation and Trp catabolism	Liver metabolism, infection, renal handling, corticosteroids, diet	Tumor enzyme/metabolite profiling; immune phenotyping
Tumor tissue	IDO1, TDO2, IL4I1 expression; Kyn or KYNA; AHR target genes	Which node is dominant in the tumor compartment	Spatial heterogeneity; stromal versus tumor-cell attribution	Multiplex imaging, spatial transcriptomics, paired plasma data
Immune-cell compartment	Treg frequency, CD8 exhaustion markers, myeloid polarization, AHR signatures	Whether pathway activity is functionally immunosuppressive	Treatment effects, timing, tissue accessibility	Tumor metabolomics and enzyme expression
Stool or mucosal samples	Indoles, fiber-responsive metabolite profiles, microbiome composition	Barrier-oriented or microbiome-driven Trp handling	Diet, antibiotics, bowel preparation, site-specific ecology	Mucosal markers, clinical phenotype, paired systemic sampling

**Table 2:** Selected therapeutic strategies engaging Trp metabolism.

Agent	Molecules	Clinical Trial	Setting	Status	Key Takeaway
Epacadostat + pembrolizumab	IDO1 + PD-1	NCT02752074	Metastatic melanoma	Completed	Phase 3 negative in biomarker-unselected melanoma
Epacadostat + pembrolizumab	IDO1 + PD-1	NCT03414229	Advanced sarcoma	Active, not recruiting	Selected sarcoma settings still being tested
BAY 2416964	AHR antagonist	NCT04069026	Solid tumors	Completed	AHR blockade is clinically feasible
IK-175 +/− nivolumab	AHR antagonist	NCT04200963	Solid tumors/urothelial carcinoma	Completed	Downstream combination strategies are testable
MK-6598 +/− pembrolizumab	Alternative catabolite program	NCT05594043	Advanced solid tumors	Completed	Noncanonical metabolite PD is entering trials

## Data Availability

No new data were created or analyzed in this study. Data sharing is not applicable.

## Abbreviations

AHR	Aryl hydrocarbon receptor
CNS	Central nervous system
CRC	Colorectal cancer
EAE	Experimental autoimmune encephalomyelitis
EMT	Epithelial–mesenchymal transition
EPIC	European Prospective Investigation into Cancer and Nutrition
FMT	Fecal microbiota transplantation
GBM	Glioblastoma
GCN2	General control nonderepressible 2 kinase
GPR35	G protein-coupled receptor 35
HCC	Hepatocellular carcinoma
IAA	Indole-3-acetic acid
IacrA	Indole-3-acrylic acid
Iald	Indole-3-aldehyde
ICI	Immune checkpoint inhibitor
IDO1	Indoleamine 2,3-dioxygenase 1
IDO2	Indoleamine 2,3-dioxygenase 2
IFNγ	Interferon-gamma
IL-22	Interleukin-22
IL4I1	Interleukin-4-induced gene 1
ILA	Indole-3-lactate
IPA	Indole-3-propionate
IpyA	Indole-3-pyruvate
Kyn	Kynurenine
KYNA	Kynurenic acid
LAT1	L-type amino acid transporter 1
NAD	Nicotinamide adenine dinucleotide
NAMPT	Nicotinamide phosphoribosyltransferase
NAPRT	Nicotinate phosphoribosyltransferase
NK cell	Natural killer cell
NKG2D	Natural killer group 2 member D receptor
NSCLC	Non-small cell lung cancer
ORR	Objective response rate
PD-1	Programmed cell death protein 1
PD-L1	Programmed death-ligand 1
PFS	Progression-free survival
QPRT	Quinolinate phosphoribosyltransferase
SLC7A5	Solute carrier family 7 member 5
TCR	T-cell receptor
TDO2	Tryptophan 2,3-dioxygenase
TME	Tumor microenvironment
TNBC	Triple-negative breast cancer
Treg	Regulatory T cell
Trp	Tryptophan
